# ESPO/ Behavioral and Social Sciences Section Symposium: Promoting Behaviors That Support Healthy Aging

**DOI:** 10.1093/geroni/igaa057.1961

**Published:** 2020-12-16

**Authors:** Elliane Irani, Briana Sprague, Luke Stoeckel

## Abstract

Maintaining healthy behaviors has been linked to positive emotional and physical health outcomes. Older adults are at a greater risk for functional decline and can benefit from the protective effects of health behaviors. The purpose of this symposium is to present and highlight: (1) innovative research linking health behaviors and health outcomes among older adults, and (2) work of emerging scholars in the Behavioral and Social Sciences (BSS) section. The papers highlight findings from descriptive studies and randomized trials testing behavioral health interventions. O’Brien and Hess describe patterns of engagement in health-promoting activities and highlight mediating and moderator factors. Fausto and colleagues report on physical activity and cognitive health benefits of a multi-level intervention focused on heart and brain health for older African American residents of public and subsidized housing. Still and colleagues assess the efficacy of a multi-component technology-based intervention on hypertension self-management in African American older adults. Nehrkorn-Bailey and colleagues report on the pilot testing of AgingPLUS, an intervention targeting attitudinal and motivational barriers to physical activity and highlight improvements in grip strength and blood pressure. Lastly, Wierenga and colleagues test an emotion regulation intervention following a cardiac event and highlight the intervention’s potential efficacy in improving mental health and physical activity. These papers underscore the importance of promoting healthy behaviors in older adults and the need for large-scale interventions that support healthy aging. As discussant, Atienza will assess the strengths and limitations of these papers, and consider how emerging scholars can contribute to the field.

